# Anthropometric Dimensions and Bone Quality in International Male Beach Handball Players: Junior vs. Senior Comparison

**DOI:** 10.3390/nu13061817

**Published:** 2021-05-27

**Authors:** Alejandro Martínez-Rodríguez, Javier Sánchez-Sánchez, Manuel Vicente-Martínez, María Martínez-Olcina, Laura Miralles-Amorós, Juan Antonio Sánchez-Sáez

**Affiliations:** 1 Department of Analytical Chemistry, Nutrition and Food Science, Faculty of Sciences, Alicante University, 03690 Alicante, Spain; maria.martinezolcina@ua.es (M.M.-O.); lma52@alu.ua.es (L.M.-A.); 2 Alicante Institute for Health and Biomedical Research (ISABIAL Foundation), 03010 Alicante, Spain; 3 School of Sport Sciences, Universidad Europea de Madrid, 28670 Madrid, Spain; 4 Faculty of Health Science, Miguel de Cervantes European University, 47012 Valladolid, Spain; mvmartinez11006@alumnos.uemc.es; 5 GDOT Research Group, Faculty of Sport, Universidad Católica de Murcia, 30107 Murcia, Spain; jasanchez419@ucam.edu

**Keywords:** body composition, team sports, exercise, athletes, bone mineral density, muscle mass, phantom, proportionality

## Abstract

Background: Beach handball is a recent team sport characterized by defensive and offensive actions on a sand surface. Scientific evidence has shown that body composition is fundamental in sports performance. The main objective of this study was to know the body composition, anthropometric characteristics, and bone mineral density of elite beach handball players. Furthermore, another purpose was to analyze the differences between categories (junior and senior) and playing position. *Methods:* A descriptive, cross-sectional study of 36 male players (18 juniors and 18 seniors) of the Spanish National Beach Handball Team was conducted. Full profile anthropometry and calcaneal ultrasound measurements were used. *Results*: Significant differences between categories (*p* < 0.05) were found in: height, body mass, arm span, BMI, muscle mass, fat mass, bone mass, skinfolds, and body perimeters. The somatotype changes depending on the playing position. Bone mineral density of the players was adequate. No significant differences were found by playing position. *Conclusions*: Senior players had a better body composition due to the presence of less fat mass than junior players. This study provides reference values of elite junior and senior beach handball players and by playing positions. This data is useful for the identification of talents and players who should be trained to improve their body composition.

## 1. Introduction

Beach handball (BH) is a sport that derives from indoor handball. This specialty became popular in Italy in the 1990s, however, it was not until the last ten years that it became a global sport [1]. BH players play on an unstable surface such as sand, which means an increase in energy expenditure and neuromuscular needs compared to indoor handball [2]. In beach handball there are various actions such as throwing, passing, jumping, blocking, running, etc. that make it an intermittent high intensity contact sport [3,4]. The different playing positions are goalkeepers, wings, specialist, pivots, and defenders [5,6].

In recent years, research has begun on this sport, finding that the main variables affecting performance are morphology, body composition and, physical and physiological characteristics [2,5,7,8,9,10,11,12]. This is because BH is a sport with defensive and offensive actions of great speed to achieve the ultimate objective of scoring a goal [13].

The specific characteristics of beach handball are frequent changes in intensity, specific skills and social factors. These aspects define the determinants of coordination, endurance, strength and cognition in this sport [14,15]. To achieve optimal performance, actions must be performed with maximum intensity [16,17]. In BH players, groupings are made by date of birth, dividing them into juniors and seniors [16]. Therefore, depending on the age category, body composition and technical abilities differ, influencing success and development as a player [16,18].

As mentioned above, numerous studies have shown that body composition and anthropometric measurements are determinant in youth and senior handball players, both in indoor and beach handball [16,18,19,20]. Many research studies have shown that optimal body composition in athletes is associated with improvements in physical performance (aerobic and anaerobic) and muscle strength [21,22,23,24,25,26]. For optimal performance of BH players, it is necessary that their weight and fat percentage are within the recommended parameters for their age group, position, and sex [6,9].

Anthropometric characteristics have been shown to be decisive in indoor handball in junior and senior teams [16,18,20,27]. In addition, a direct influence between body composition and performance tests has been observed [6,28]. Milanese et al. [29] evaluated body composition as a function of playing position and found some significant differences between players. Body mass index (BMI) and indirect estimates of fat mass were commonly used to analyze body composition [16]. However, these methods have been discarded due to their limitations, as BMI is not only related to fat mass, but also to lean mass [30]. Therefore, in recent years, higher-quality investigations use methods such as dual X-ray densitometry (DXA) and full anthropometry [30].

Whole body composition as a whole includes body size and the proportion of body compartments. Body composition is usually analyzed through anthropometric measurement of weight, skinfolds, circumferences, diameters, heights and BMI [31]. Body size is of great importance for throwing in attack or blocking in defense, achieving higher ball velocity in jump throwing also having a strong positive effect on throwing performance and isometric strength [14,32]. The presence of a high percentage of fat is associated with multiple diseases and inflammation, so it has negative health consequences [6]. The optimal composition of athletes is framed by small amounts of fat mass and high amounts of muscle mass [33,34]. The specific percentage for adequate performance depends on the sporting position [33,35,36].

Forward players have displayed more favorable body composition results than other playing positions in indoor handball [37]. The morphology and composition of the upper limbs are fundamental aspects in beach handball. The best elite indoor handball players have shown higher values for humerus amplitude and hand length and width, these traits are found in the upper extremities and cannot be modified by training [38].

Therefore, it can be seen how the assessment of body composition is a fundamental aspect in sport due to its relation with performance and injury prevention, highlighting the importance of fat and muscle mass content [39,40]. It has been shown that fat mass, as opposed to muscle mass, is dead weight for jumping and sprinting, actions frequently performed in BH [39,41].

Bone mass is another relevant component to consider. The assessment of bone mineral density (BMD) is a measure that informs us about bone condition and strength. BMD is inversely related to the occurrence of fractures [42]. Skeletal injuries are rare in athletes, but their occurrence can have serious consequences for the athlete’s health and professional life [43]. Physical exercise plays an important role in bone mass during growth. Beach handball is a sport that involves high mechanical stress on the lower limbs due to high intensity running, jumping and landing, causes osteogenic reactions [42,44].

Despite the increase of research in this sport in recent years, data about physical characteristics and bone mineral density in elite BH players are scarce [9]. Knowledge of the anthropometric profiles of these players is necessary to be able to identify the most important aspects which will have to be improved in order to achieve optimal sport performance.

The main objective of this research was to describe the body composition of elite male junior and senior BH players. The specific objectives were: (a) to know the body composition and bone mineral density of elite BH players by categories and playing position (b) to analyze the differences in body composition according to categories. The initial hypothesis was that body composition would be different between youth and senior players; and that the players with the best body composition would be the forwards.

## 2. Materials and Methods

### 2.1. Study Design

A descriptive, cross-sectional study was used to analyze the body composition and bone mineral density of male beach handball players, measured by anthropometry and calcaneal ultrasound, respectively. The research was conducted in accordance with the ethical standards recognized by the Declaration of Helsinki. The study was approved by the Ethical Committee of Alicante University (UA-2019-04-09).

### 2.2. Subjects

The study sample consisted of 36 male beach handball players (18 junior; 16.7 ± 0.46 years and 18 seniors; 25.0 ± 5.19 years). All of them were professional players of the National Beach Handball Team of the Royal Spanish Handball Federation, therefore, they represent the elite of BH players. The sample is divided into goalkeepers, wings, specialists, pivots, and defenders. All players received information about the objectives of the research, the experimental protocol, and the study procedures. Each of the participants signed the informed consent document. In the case of underage players, parents or legal tutors gave permission. Anonymity was preserved for all participants.

### 2.3. Anthropometric Data

Anthropometric variables were measured for each subject. For this purpose, full profile was developed, following the standard protocol of the International Society for the Advancement of Kinanthropometry (ISAK) [45].

All measurements were performed by the same investigator, an ISAK level 2 anthropometrist. The mean technical error was less than 1% for perimeters, circumferences, lengths, and heights and less than 5% for skinfolds. All measurements were performed on the first day of the concentration, under basal conditions, in the same location and at room temperature (22 ± 1 °C).

The following anthropometric material was used as approved and previously calibrated: wall measuring rod (accuracy, 1 mm); digital scale (BC545N, Tanita, Tokyo, Japan; accuracy, 100 g); metallic, narrow, and an inextensible measuring tape (Lufkin, TX, USA; accuracy, 1 mm); small bone diameter pachymeter (Smartmet, Jalisco, Mexico; accuracy, 1 mm); skinfold caliper (Harpenden, UK; accuracy, 0.2 mm), complementary material (demographic pencil to mark the players) and anthropometric bench measuring 40 × 50 × 30 cm.

Height and seated height were determined using a mobile anthropometer (Seca 213, SECA Deutschland, Hamburg, Germany) to the nearest millimeter, with the participant’s head maintained in the Frankfort Horizontal Plane position. The length of wingspan was measured with an arm span meter (Smartmec, Zapopan Jalisco, México), made with a steel tape 5 m long and 18 mm wide, with an accuracy of 1 mm. Eight skinfolds were collected (subscapular, tricipital, bicipital, iliac crest, supraspinal, abdominal, anterior thigh and medial calf); 13 perimeters (head, neck, thorax, relaxed arm, contracted arm, forearm, wrist, waist, hip, thigh 1 cm from the glute, medial thigh, maximum leg and minimum ankle); 9 bone diameters (biacromial, anteroposterior abdominal, biliocrestal, transverse thorax, anteroposterior thorax, biepicondylar humerus, bi-styloid and bicondylar femur, bimalleolar); 8 lengths and heights (foot length, acromiale-radiale, radiale- stylion, midstylion-dactylion, iliospinale height, trochanterion height, trochanterion-tibiale laterale, tibiale laterale height, tibiale medial-sphyrion tibiale). The sum of 6 skinfolds was also computed (subscapular, triceps, supraspinale, abdominal, front thigh and medial calf).

Body composition was calculated using the following models: fat mass was estimated using the methods of Withers et al. [46] and Faulkner [47]. Muscle and bone masses were determined using the methods of Lee et al. [48] and Rocha [49], respectively. According to the Spanish Committee of Kinanthropometry, these methods are the most suitable for high performance players [49].

### 2.4. Somatotype

The mean somatotype and classification were determined using the anthropometric method of Heath and Carter [50] and its classification [51]. The somatotype is defined as the quantification of the shape and composition of the human body. It is represented by three components: (1) endomorphy (2) mesomorphy and (3) ectomorphy. Each component was calculated with its corresponding formulas [52].

### 2.5. Anthropometric Dimensions—Proportionality

Proportionality analysis were performed using the Phantom stratagem; a bilaterally symmetrical, bilaterally symmetrical, conceptually modeled, reference human derived from male and female reference data, proposed and revised by Ross and Ward [53]. Each variable was adjusted to the Phantom size using z-score = (1/s) × v × [(170.18/h)^d^ − *p*]; where z = proportionality value, v = size of any given variable, 170.18 = Phantom stature constant, h = subject’s stature, d = dimensional exponent, P = Phantom value for variable v, and s = Phantom standard deviation value for variable based on an hypothetical universal human population. The z-values have a mean of 0, so a z-value of 0.0 indicates that the variable v is proportionally equal to that of the Phantom; a z-value greater than 0.0 means that the subject is proportionally greater than the Phantom for the variable v; otherwise, a z-value less than 0.0 shows that the subject is proportionally less than the Phantom for that variable [53].

### 2.6. Bone Quality

A heel ultrasound densitometer (Achilles EXP II, GE Healthcare, Chicago, IL, USA) was used to measure the bilateral calcaneus of each subject. Quality assurance was performed before the first measurement, by calibrating the device on a dedicated phantom supplied by the manufacturer. In addition, to ensure good contact, an ultrasound gel was applied. Broadband ultrasound attenuation (BUA) and speed of sound (SOS) were directly measured during each ultrasonographic evaluation. The calcaneal stiffness index was calculated using the following formula, previously used in other studies [54]:

Calcaneal stiffness (A.U.) = (0.67 − BUA + 0.28 − SOS) − 420

The elastic resistance of the bone is measured by the variable SOS, while the loss of ultrasound energy that occurs by absorption or scattering (and correlates with bone density) is evaluated by the variable BUA. By a combination of SOS and BUA, stiffness is achieved.

### 2.7. Statistical Analyses

To show the characteristics of the participants, descriptive statistics were made for all variables (Mean ± SD). To test the normality of the sample Kolmogorov-Smirnov, Shapiro-Wilk and Levene’s test were applied. Analysis of variance (ANOVA), with Bonferroni post hoc comparisons to identify differences in basic anthropometric and demographic characteristics between players. Analysis of covariance (ANCOVA) with the correction of Bonferroni was used to compare differences between age groups (junior vs. senior), only the variables related to body composition were adjusted by BMI. The Somatotype Attitudinal Distance (SAD) was used to compare somatotype group means of junior and senior players. Statistical significance was set at *p* < 0.05. Cohen’s d was used as a measure of the effect size (ES) of the differences between junior and senior players. The thresholds stipulated by Cohen [55] were considered; small (d = 0.2), moderate (d = 0.6), large (d = 1.2), very large (d = 2.0) and extremely large (d = 4.0). Mean differences in the chosen anthropometric characteristics, body composition and somatotype components of the players between playing positions were tested using a general linear model with a Tukey’s post hoc test (*p* < 0.05) and using BMI as a covariate. All statistical analysis were performed using the Jamovi 1.1.3.0 software (The jamovi project, Sydney, Australia). The z-phantom scores were obtained from Excel and were represented in graphic form.

## 3. Results

A total of 32 male beach handball players participated in this study: 50% juniors and 50% seniors. Table 1 shows the basic anthropometric measurements. Mean weight is 78.1 ± 12.2 kg and 90.1 ± 13.4 kg for juniors and seniors, respectively. Height is 181 ± 5.90 cm for juniors and 188 ± 7.73 cm for seniors. Senior players show higher values, presenting significant differences (*p* < 0.05) in all variables, including arm span, which is 184 ± 7.45 cm for junior and 193 ± 9.35 cm for senior. In addition, generally the effect sizes were moderate to high. Due to these differences, BMI will be used as a covariate to analyze all the differences in the rest of the variables analyzed.

Table 2 shows the body composition values (fat mass, muscle mass, bone mass, residual mass) and Table 3 the SOS, BUA and Stiffness values measured by ultrasound of all players, separated by age group: senior vs. junior. Statistically significant differences were observed in muscle mass (*p* < 0.01), fat mass measured by the Withers equation and bone mass (*p* < 0.05). In all variables, the results were higher in seniors, except in the percentage of fat calculated with the Withers formula.

**Figure 1:**
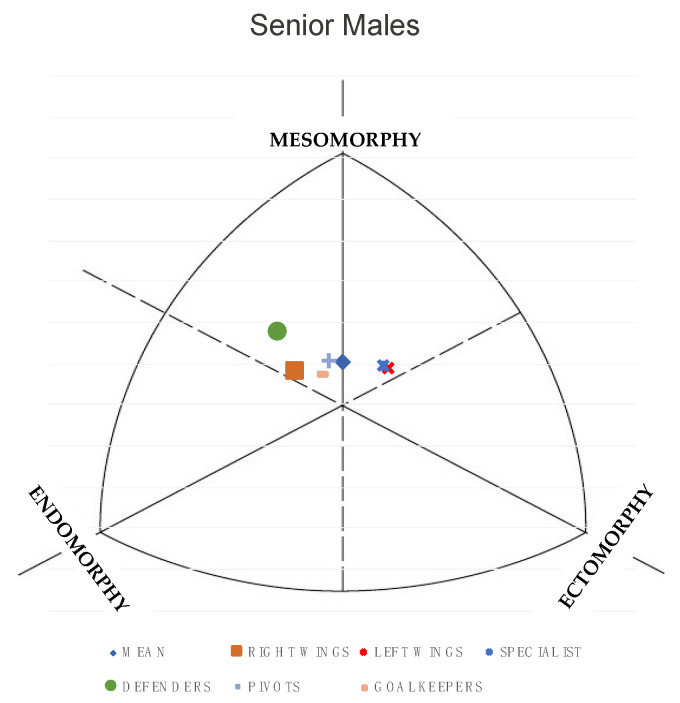
Somatotype distribution of elite male junior handball players.

**Figure 2:**
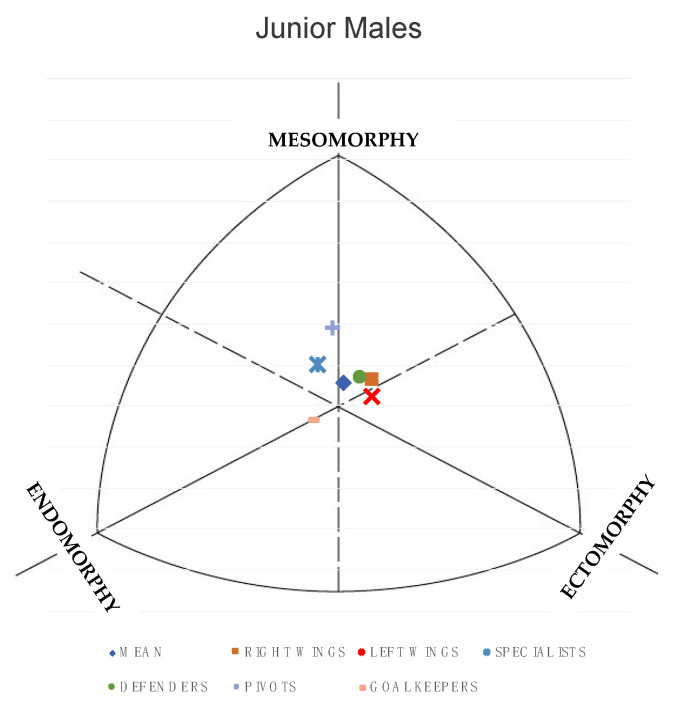
Somatotype distribution of elite male senior beach handball players.

The differences in somatotype, ponderal index and Somatotype Attitudinal Distance (SAD) between juniors and seniors are shown in Table 4. Significant differences were observed in the endomorphic (*p* < 0.05), ectoomorphic (*p* < 0.05) and ponderal index (*p* < 0.05) components. In all 3 variables the values are higher in junior players. As shown in Figure 1 and Figure 2, the mean somatotype for junior and senior male players can be defined as balanced mesomorph (2.6-3.7-2.7) and (2.8-3.4-2.9), respectively.

However, if somatotype is analyzed as a function of playing position, as shown in the figure, the trends are different. For juniors, goalkeepers present a balanced endomorph somatotype, while right and left wings and defenders are mesomorph-ectomorph. In the senior category, defenders and right wings have a mesomorphic-endomorphic somatotype, while specialists and left wings tend to have a mesomorphic-ectomorphic somatotype.

Table 5 There are significant differences in some skinfolds such as triceps (*p* < 0.01), biceps (*p* < 0.05), front thigh (*p* < 0.05) and medial calf (*p* < 0.01), as well as in the sum of 6 skinfolds (*p* < 0.05). There was a general tendency for senior players to have lower skinfold values. Overall effect sizes were moderate to large.

The results in Table 6 show the descriptive statistics and the differences of the selected variables between players according to their playing position. Significant differences (*p* < 0.05) were only observed between goalkeepers and wings in the variable SOS.

Results shows that there has been a slight difference in some of the variables analyzed between juniors and seniors. The comparison between goalkeepers and specialists gave values of *p* = 0.076 and ES = 1.57, between goalkeepers and pivots of *p* = 0.067 and ES = 1.68; therefore, the ES in both cases were high. For the Stiffness variable, between the goalkeepers and the wings the values were, *p* = 0.057 and ES = 1.64, so there were also differences.

**Figure 3:**
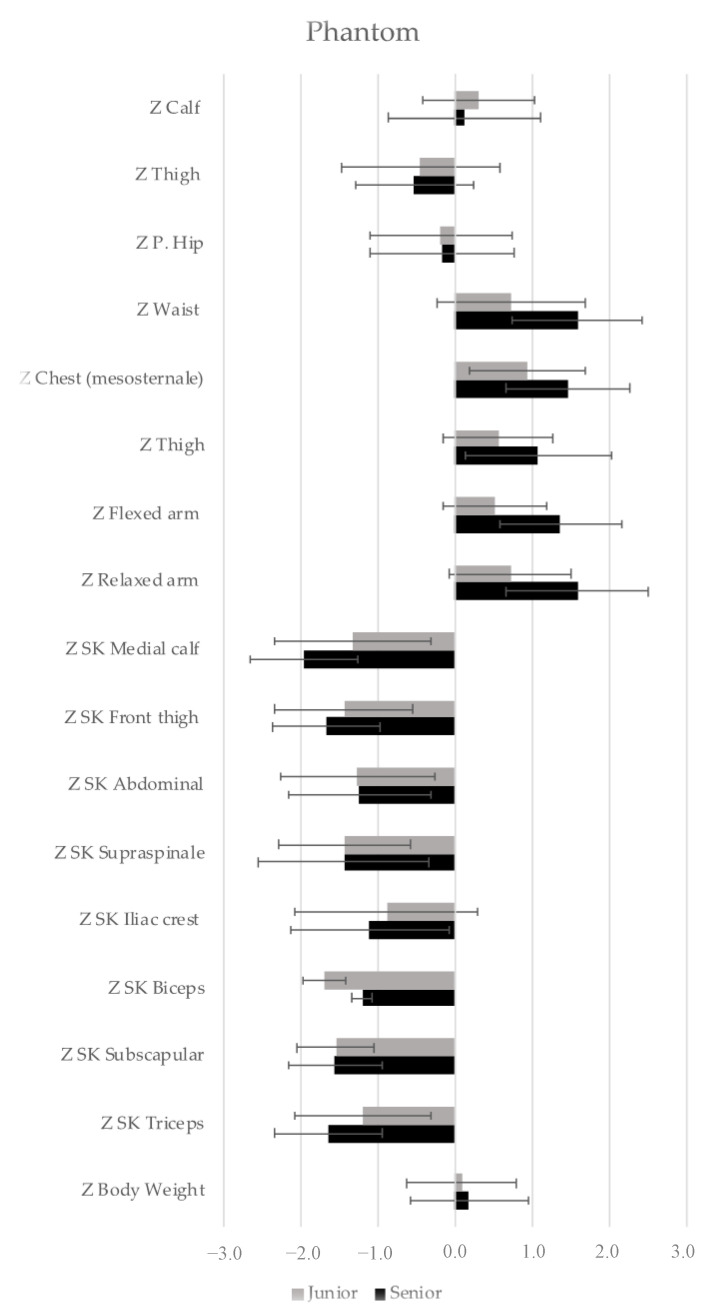
Representation of the proportionality with respect to the phantom. SK = Skinfolds. Data are presented as mean and standard deviation.

Figure 3 shows the anthropometric dimensions, as proportionality profiles of the junior and senior players. Goalkeepers have been excluded due to the particularity of their playing position. After analysis, significant differences were observed in some variables such as Z skinfold calf (*p* = 0.032; ES = 0.744; MD = 0.661), Z relaxed arm (*p* = 0.041; ES = −0.704; MD = −0.691), Z Flexed arm (*p* = 0.005; ES = −0.990; MD = −0.726); Z forearm (*p* = 0.049; ES = −2.94; MD = −0.543); Z Chest (mesosternale) (*p* = 0.030; ES = −0.756; MD = −0.591) and waist circumference (*p* = 0.019; ES = −0.825; MD = −0.851). In all the variables described, the results are lower for junior players.

## 4. Discussion

The aim of this research was to analyse the anthropometric profile, body composition and somatotype of elite BH players according to category (junior vs. senior) and playing positions. The results showed significant differences between junior and senior categories in several components such as kg of muscle mass, kg of fat mass and kg of bone mass, as well as skinfolds (triceps, biceps, thigh, calf and sum of six skinfolds) and perimeters (arm, contracted arm and waist). However, no differences in body composition and anthropometric profile by playing position have been found. Other studies have investigated body composition in Spanish senior elite BH players [9,12]. However, the study by Zapardiel et al. [12] only analysed weight, height and BMI. On the other hand, Pueo et al. [9] did study the anthropometric profile and somatotype, but the study was of doubtful reliability due to the small sample size used. Another of identified weaknesses in this study is the grouping of players according to playing position: goalkeepers, front players and back players; categorising wings-specialists and pivots-defenders as the same, an aspect that undermines the principle of specificity in training. In the present research, each specific position has been studied individually: goalkeepers, wings, specialists, pivots, and defenders. So far, no studies have been conducted to examine the anthropometric, BMD and somatotype characteristics of elite junior BH players. One strength of this scientific paper is that it provides a frame of reference for junior elite BH players.

The junior elite BH players showed a mean height of 181 ± 5.90 cm and a body mass of 78.1 ± 12.2 kg, while the seniors had a height of 188 ± 7.73 cm and weight of 90.1 ± 13.4 kg. The differences found are due to the different stages of development of the junior vs. senior players. These results are slightly higher than the ones obtained in similar studies for senior players where the mean height results were: 187.4 ± 8.2 cm [9] and 187.5 ± 7.5 cm [12]; and mean weights of: 85.2 ± 11.3 kg [9] and 87.0 ± 9.5 kg [12]. Thus, according to the results of the present study, senior BH players are moderately taller and heavier than the players analysed in other studies. Consequently, the BMI presented in the senior players of the study (25.4 ± 2.50 kg/m^2^), is higher than those presented in the studies of Pueo et al. [9] and Zapardiel et al. [12] being 24.2 ± 2.5 kg/m^2^ and 24.9 kg/m^2^, respectively.

The body composition of the players studied showed significant differences in muscle mass (kg), fat mass, measured with Wither’s formula (kg), and bone mass (kg). This distinction can be explained by the significant difference between juniors and seniors in total body mass. The juniors showed a muscle mass of 42.5 ± 3.45%, while the seniors had a percentage of 42.8 ± 4.03%. The data was similar to the one obtained from the research carried out by Pueo et al. [9] in which the muscle mass results were 42.7 ± 2.6%.

Regarding fat mass, both studies used the Withers formula for its calculation and for this reason, they are comparable. The present study obtained a fat mass in juniors of 13.8 ± 6.73% and in seniors of 13.1 ± 5.03%. The senior players who participated in the study by Pueo et al. showed a fat percentage of 11.7 ± 3.9% [9]. These results were lower than those presented by the players in our study, possibly due to the fact that the players were in better physical shape, also because the data collection could take place at a different time of the season. Comparing these results with the indoor modality [56], players playing on court have lower values of fat mass (11.3 ± 2.4%) than those of the present study and other BH studies [9].

Significant differences were found between juniors and seniors in skinfolds. The sum of 6 skinfolds presented by the juniors was 71.9 ± 34.8 mm, while for seniors was 69.1 ± 27.3 mm. These results coincided with the data obtained for fat mass and were similar to the results of other studies such as Pueo et al. [9]; 62.9 ± 24.1 mm. The present data was lower than those found in elite indoor handball players (77.2 ± 27.5 mm) [57]. Therefore, it can be concluded that skinfold measurements in indoor handball are higher, i.e., with more subcutaneous fat, than in BH [9].

Regarding bone mass and BMD, the junior players had 15.5 ± 1.34% bone mass and BUA, SOS and Stiffness values of 131 ± 10.3 dB/MHz, 1640 ± 33 m/s and 127 ± 14.5 (A.U), respectively. On the other hand, senior players obtained a bone mass of 14.9 ± 1.07% and BUA, SOS and Stiffness of 131 ± 9.24 dB/MHz, 1657 ± 33.1 m/s and 133 ± 11.9 (A.U). Pueo et al. [9] obtained similar results in senior BH players 15.7 ± 1.6%, however, they did not analyse BMD, so BUA, SOS and Stiffness results cannot be compared with other BH players due to the lack of studies. Studies conducted in Spanish senior population yield lower results in BUA than those presented in the current research (93.42 ± 18.38 dB/MHz) [58] and (84.5 ± 18.4 dB/MHz) [59]. The results found for SOS were also lower than the ones showed on the paper (1567.5 ± 33.3 m/s) [59]. In regard to BMD assessment in juniors, other populations have obtained a BUA of 89.46 ± 14.41 dB/MHz and an SOS of 1503.54 ± 13.45 m/s [60]. These differences from the results of the present investigation are due to the fact that exercise is associated with an increase in bone mineral density [61].

The junior and senior elite BH players presented a balanced mesomorphic somatotype (2.79-3.40-2.91 and 2.69-3.73-2.70, respectively). These results were similar to those presented in the study by Pueo et al. [9], although the mesomorphy value was lower (2.6-4.4-2.7).

In respect of variations in anthropometric profile and body composition between playing positions, the present study found no significant differences except in SOS. However, Pueo et al. [9] found variations to be considered in height, weight and wingspan, but not in the rest of the components. These differences can be explained by the small sample size analysed in the Pueo et al. [9] research.

The body proportionality profiles of the BH players were similar to each other, although the junior players showed lower results. Significant differences were found in calf skinfold, flexed arm, forearm, thorax and waist circumferences. To the authors’ knowledge, this is the first study to assess proportionality in beach handball players, so comparisons with other similar studies cannot be made. This research will be useful to confirm the proportionality values through further studies.

The present study was not exempt from limitations, one of the limitations may be that both body composition and BMD values were studied with full anthropometry and calcaneal ultrasound due to the feasibility of the research. Another limitation that should make the results between playing positions to be interpreted with caution, is that there were not the same number of players in the different categories. Future investigations should be carried out on a sufficient and equal number of players per playing position and with gold standard instruments such as DXA. Furthermore, these data refer to Caucasian players, so for other populations these data should be interpreted with caution.

However, considering the above limitations, the results of this research are of great relevance since they incorporate junior category data, so far not studied in BH, and also provide more complete information and a larger number of samples than previous research carried out in BH players [9,12]. These data should be useful for the recruitment and selection of players with the optimum profile for performance in beach handball and the detection of talent in young players.

## 5. Conclusions

The anthropometric profile, as well as body composition and somatotype, play a fundamental role in the optimal performance of elite BH players. This research examined the differences in male BH players by categories (junior vs. senior) and by playing positions (goalkeepers, wings, specialists, pivots, and defenders).

Differences between age groups were found in height, body mass, arm span, BMI, muscle mass, fat mass, bone mass, skinfolds, and body perimeters. Body composition was more optimal in senior players due to the presence of less fat mass. The mean somatotype of both categories was mesomorph balanced. No significant differences were found in anthropometric and body characteristics according to playing position.

The data provided by this study is considered of great interest to compare and obtain a reference for elite BH players. Future research should focus on analyzing these parameters on a larger number of players per playing position and to achieve decisive references using more precise body estimation methods such as DXA.

## Figures and Tables

**Table 1:** Basic anthropometric and demographic characteristics of the sample.

Variable	Junior (*n* = 18)	Senior (*n* = 18)	ANOVA			
	Mean ± SD	Mean ± SD	Mean Difference	t	*p*	Cohen’s d
Age (years)	16.7 ± 0.46	25.0 ± 5.19	−8.28	−6.75	<0.001	2.25
Body height (cm)	181 ± 5.90	188 ± 7.73	−7.65	−3.34	0.002	1.11
Body mass (kg)	78.1 ± 12.2	90.1 ± 13.4	−12.0	−2.82	0.008	0.94
Arm span (cm)	184 ± 7.45	193 ± 9.35	−8.84	−3.14	0.004	1.05
BMI (kg/m^2^)	23.9 ± 2.82	25.4 ± 2.50	−1.47	−1.65	0.107	0.55

SD: Standard Deviation; BMI: Body Mass Index; Cohen’s d (Effect Size); Mean differences were significant at *p* < 0.05.

**Table 2:** Descriptive data on body composition and differences between senior and junior.

Variable	Junior	Senior	Ancova (Adjusting by BMI)			
	Mean ± SD	Mean ± SD	Mean Difference	t	*p*	Cohen’s d
Muscular mass (kg)	32.9 ± 3.38	38.2 ± 3.87	−3.88	−4.22	<0.001	1.460
Muscular mass (%)	42.5 ± 3.45	42.8 ± 4.03	−1.71	−1.86	0.072	0.644
BFM Withers (kg)	11.4 ± 7.17	12.2 ± 6.23	3.32	2.66	0.012	0.920
BFM Withers (%)	13.8 ± 6.73	13.1 ± 5.03	2.31	1.88	0.070	0.650
BFM Faulkner (kg)	10.4 ± 4.23	12.1 ± 4.19	1.06	1.52	0.137	0.528
BFM Faulkner (%)	12.9 ± 3.39	13.2 ± 3.01	0.260	0.36	0.722	0.124
Bone mass (kg)	12.0 ± 1.28	13.3 ± 1.58	−0.845	−2.12	0.041	0.736
Bone mass (%)	15.5 ± 1.34	14.9 ± 1.07	0.0923	0.35	0.727	0.122
Residual mass (kg)	22.9 ± 4.49	26.5 ± 5.68	−1.30	−1.33	0.191	0.462
Residual mass (%)	29.1 ± 2.02	29.2 ± 2.30	0.553	0.85	0.399	0.296

SD: Standard Deviation; BFM: Body Fat Mass; t: t student; Mean differences were significant at *p* < 0.05.

**Table 3:** Descriptive data on bone quality and differences between senior and junior.

Variable	Junior	Senior	Ancova (Adjusting by BMI)			
	Mean ± SD	Mean ± SD	Mean Difference	t	*p*	Cohen’s d
BUA (dB/MHz)	131 ± 10.3	131 ± 9.24	1.43	0.43	0.672	0.148
SOS (m/s)	1640 ± 33.0	1657 ± 33.1	−11.9	−1.07	0.293	0.370
Stiffness (A.U)	127 ± 14.5	133 ± 11.9	−2.39	−0.54	0.537	0.188

SD: Standard Deviation; BUA: Broadband ultrasound attenuation; SOS: Speed of sound; t: t student; Mean differences were significant at *p* < 0.05.

**Table 4:** Somatotype components and difference between male and female players.

Variable	Junior	Senior	Ancova (Adjusting by BMI)			
	Mean ± SD	Mean ± SD	Mean Difference	t	*p*	Cohen´s d
Endomorphy	2.79 ± 1.32	2.69 ± 1.16	0.104	0.251	0.033	0.770
Mesomorphy	3.40 ± 0.97	3.73 ± 1.00	−0.021	−0.07	0.941	0.026
Ectomorphy	2.91 ± 1.18	2.70 ± 0.96	−0.340	−2.39	0.023	0.826
Ponderal index	43.0 ± 1.61	42.7 ± 1.30	−0.464	−2.39	0.023	0.826
SAD	3.30 ± 3.01	2.77 ± 1.76	0.317	0.369	0.715	0.128

SAD: Somatotype Attitudinal Distance; SD: Standard Deviation; t: t student; Mean differences were significant at *p* < 0.05.

**Table 5:** Descriptive data on skinfolds, circumferences, diameters, and the differences between junior and senior are presented in Table 4.

		Junior	Senior	Ancova (Adjusting by BMI)			
	Variable	Mean ± SD	Mean ± SD	Mean Difference	t	*p*	Cohen’s d
Skinfolds	Triceps (mm)	10.7 ± 4.96	9.09 ± 3.65	3.52	3.75	<0.001	1.300
	Subscapular (mm)	9.87 ± 3.83	10.8 ± 3.98	0.416	0.392	0.697	0.136
	Biceps (mm)	6.08 ± 4.21	5.11 ± 1.84	2.01	2.16	0.038	0.748
	Iliac crest (mm)	17.0 ± 9.01	17.0 ± 7.97	3.61	1.89	0.067	0.655
	Supraspinale (mm)	9.53 ± 5.36	10.2 ± 5.72	1.58	1.22	0.230	0.423
	Abdominal (mm)	16.1 ± 8.73	18.1 ± 8.04	1.45	0.750	0.458	0.260
	Front thigh (mm)	15.4 ± 8.50	13.5 ± 6.67	4.99	2.72	0.010	0.944
	Medial calf (mm)	10.3 ± 5.41	7.31 ± 3.50	4.82	4.38	<0.001	1.520
	6 skinfolds (mm)	71.9 ± 34.8	69.1 ± 27.3	16.8	2.59	0.014	0.898
Girths	Relaxed arm (cm)	30.6 ± 3.12	33.7 ± 2.50	−1.75	−3.17	0.003	1.100
	Flexed arm (cm)	32.6 ± 2.37	35.9 ± 2.38	−2.28	−4.15	<0.001	1.440
	Thigh (cm)	54.1 ± 6.27	56.2 ± 3.83	0.523	0.616	0.542	0.214
	Calf (cm)	37.8 ± 2.77	39.1 ± 2.76	−0.077	−0.139	0.890	0.048
	Waist (cm)	79.5 ± 5.72	87.1 ± 6.57	−4.71	−4.20	<0.001	1.460
	Hip (cm)	99.7 ± 8.85	104 ± 6.32	−0.335	−0.289	0.775	0.100
Breadths	Humerus (cm)	7.13 ± 0.33	7.33 ± 0.31	−0.0902	−1.06	0.297	0.367
	Stylion (cm)	5.54 ± 0.37	5.79 ± 0.34	−0.154	−1.39	0.173	0.483
	Femur (cm)	9.56 ± 0.55	9.76 ± 0.53	0.0366	0.322	0.750	0.111

SD: Standard Deviation; t: t student; Mean differences were significant at *p* < 0.05.

**Table 6:** Position-related differences in selected anthropometric characteristics, body composition and somatotype components of male and female players.

Variable	Goalkeepers (*n* = 6)	Wings (*n* = 12)	Specialists (*n* = 6)	Pivots (*n* = 5)	Defenders (*n* = 7)	ANOVA		
	Mean ± SD	Mean ± SD	Mean ± SD	Mean ± SD	Mean ± SD	*F*	*p*	*ηp2*
Body height (cm)	187 ± 3.11	180 ± 7.27	185 ± 12.2	188 ± 6.05	186 ± 6.50	0.643	0.636	0.079
Body mass (kg)	88.9 ± 10.9	75.1 ± 10.7	84.9 ± 15.6	92.9 ± 13.9	88.6 ± 15.3	0.540	0.708	0.067
Arm span (cm)	190 ± 6.22	184 ± 7.88	193 ± 16.7	193 ± 6.82	189 ± 6.00	0.709	0.592	0.086
BMI (kg/m^2^)	25.4 ± 3.14	23.1 ± 2.22	24.6 ± 2.51	26.1 ± 2.51	25.5 ± 2.93			
6 skinfolds (mm)	81.6 ± 40.1	61.2 ± 24.9	69.9 ± 28.0	75.0 ± 30.2	74.3 ± 38.7	0.526	0.718	0.065
Endomorphy	3.37 ± 1.77	2.45 ± 0.992	2.54 ± 1.05	2.67 ± 0.796	2.92 ± 1.52	1.23	0.321	0.140
Mesomorphy	3.19 ± 1.28	3.39 ± 0.975	3.68 ± 1.14	3.93 ± 0.589	3.85 ± 0.920	0.799	0.535	0.096
Ectomorphy	2.69 ± 1.46	3.20 ± 0.992	2.82 ± 1.13	2.38 ± 0.759	2.51 ± 0.978	0.682	0.610	0.083
MM (%)	40.6 ± 3.12	44.0 ± 3.91	43.2 ± 4.54	42.1 ± 3.68	41.9 ± 2.89	0.507	0.731	0.063
BFM Withers (%)	15.9 ± 8.08	11.7 ± 4.57	13.0 ± 5.09	14.1 ± 5.59	14.0 ± 7.23	0.622	0.650	0.077
BFM Faulkner (%)	14.7 ± 4.13	12.1 ± 2.59	12.5 ± 2.53	13.3 ± 2.55	13.3 ± 4.14	1.17	0.346	0.135
SOS (m/s)	1616 ± 24.1 #	1651 ± 33.5 #	1658 ± 27.6	1669 ± 37.9	1649 ± 31.5	3.11	0.030	0.293
BUA (dB/MHz)	129 ± 8.23	127 ± 10.6	139 ± 8.57	132 ± 6.31	132 ± 9.97	1.370	0.268	0.154
Stiffness (A.U)	119 ± 11.2	128 ± 13.0	137 ± 12.2	135 ± 11.4	130 ± 14.3	2.25	0.087	0.231

SD: Standard Deviation; BFM: Body Fat Mass; MM: Muscular mass; BUA: Broadband ultrasound attenuation; SOS: Speed of sound; t: t student; Mean differences were significant at *p* < 0.05; #: statistical significance between goalkeepers and wings.

## Data Availability

The data presented in this study is available on request from the corresponding author. The data are not publicly available due to is personal health information.
